# Factors associated with post NICU discharge exclusive breastfeeding rate and duration amongst first time mothers of preterm infants in Shanghai: a longitudinal cohort study

**DOI:** 10.1186/s13006-022-00472-x

**Published:** 2022-05-02

**Authors:** Xin Jiang, Hui Jiang

**Affiliations:** Nursing Department, Shanghai First Maternity and Infant Hospital, School of Medicine, Tongji University, No 2699, West Gaoke Road, Pudong New Area, Shanghai, 200092 China

**Keywords:** Determinants, Exclusive breastfeeding duration, Preterm infants separated from their mothers, After discharge

## Abstract

**Background:**

Breastfeeding is crucial for the preterm infants. Breast milk is not only food but also medicine. Few studies have focused on the longitudinal effects of exclusive breastfeeding outcome of preterm infants separated from their mothers after discharge, especially in Shanghai. We aimed to examine the exclusive breastfeeding rate and duration amongst first time mothers of preterm infants after discharge and its determinants.

**Methods:**

Analyses were based on 500 preterm infants separated from their mothers in a tertiary maternity and infant-specialized hospital in Shanghai from September 2018 to September 2019.The Socio-demographic characteristics, breastfeeding knowledge questionnaire, breastfeeding self-efficacy short form scale, Edinburgh postpartum depression scale and breastfeeding family support scale were used for the investigation and the exclusive breastfeeding rate of premature infants was followed up on 1 month, 3 months and 6 months after discharge. The changing trend of breastfeeding knowledge, breastfeeding self-efficacy, postpartum depression and family support were measured by ANOVA at different stages. Using the chi-square test and multiple logistic regression, factors impacting the breastfeeding rate of preterm infants at three time intervals after discharge were investigated. The Kaplan Meier survival curve and cox regression model were used to analyze the determinants of exclusive breastfeeding duration of premature infants after discharge.

**Results:**

Exclusive breastfeeding rates were 19.0, 17.2 and 10.4% at 1 month, 3 months and 6 months after discharge of preterm infants, respectively. The average length of exclusive breastfeeding duration was(3.69 ± 1.80)months. Finally, type of delivery (adjusted odds ratio [AOR] 1.564; 95% confidence interval [CI] 0.513,3.116), gestational age(AOR 0.612, 95% CI 0.236, 3.418), maternal family support (AOR 6.125,95% CI 6.359, 98.452) were discovered to be independent predictors on the exclusive breastfeeding rate at 6 months after preterm infants were discharged. Through the cox regression model, we found that a maternal planned pregnancy (HR 0.681, 95%CI 0.531,0.873), delivering breast milk during hospitalization (HR 0.797, 95%CI 0.412,2.288), NICU feeding mode during hospitalization (HR 1.221, 95%CI 0.128,1.381) and family support (HR 0.561, 95%CI 0.004,2.428) were significantly associated with the exclusive breastfeeding duration after discharge.

**Conclusions:**

The exclusive breastfeeding outcome of premature infants was affected by many factors, so we should focus on the three levels of individual, family, society and design targeted intervention measures to increase the exclusive breastfeeding rate and prolong exclusive breastfeeding duration.

## Background

Breastmilk provides the optimal nutrition for infants, protects against infection, promotes long-term health, moreover it is a crucial component of public health, especially for preterm infants (gestational age < 37 weeks) [1]. Breastfeeding has immunological, nutritional and neurodevelopmental benefits for preterm infants [2]. For example, breast milk protects preterm infants from necrotizing enterocolitis, bronchopulmonary dysplasia and late-onset sepsis [3, 4]. The WHO estimates that the global preterm birth rate was 9.8% (uncertainty interval (UI): 8.3–10.9%) in 2000, 11.1% (UI: 9.1–13.4%) in 2010 and 10.6% (UI: 9.0–12.0%) in 2014 [5, 6].

Preterm infants may have serious cardiovascular or nervous system complications after birth. Relevant studies have pointed out that the probability of death of preterm infants who weigh less than 500 g is as high as 85% [7–9]. Dysphagia and the insufficient oral sucking power of preterm infants increase the risk of breastfeeding failure [10]. After the implementation of China’s universal two-child policy in 2016,the preterm birth rate increased due to an enlarging cohort of pregnant women of advanced reproductive age [11]. Because of their immature gestational age and organs, premature infants are more likely to require separation from their mothers physically in China [12]. The parents may vacillate between separation and intimacy emotionally, they felt close as they became autonomous, made decisions and provided care for their infants under careful supervision. They left their infants’ bedsides reluctantly because this created a strong sense of separation [13].

The separation of mother and infant refers to instances where the newborn is sent to the neonatal intensive care unit (NICU) for observation and treatment due to congenital immaturity or disease after birth, resulting in the physical and psychological separation of the mother and the baby and is also an important factor leading to breastfeeding failure [13]. Breastfeeding plays a key role in the prognosis of preterm infants. For preterm infants, breastfeeding can not only meet their growth and development needs but also play a therapeutic role [14]. Breastfeeding can effectively prevent necrotizing enterocolitis in newborns [3], ROP (retinopathy of prematurity) and other serious complications [3, 4] and can reduce the mortality of premature infants [15]. Breastfeeding failure will lead to the low immunity and a higher incidence rate of chronic diseases in premature infants, which will trigger the short-term and long-term health problems of premature infants [1].

At present, given the breastfeeding failure of premature infants, the health and medical departments have issued many relevant supporting policies, including the 10 steps to successful breastfeeding implemented by the World Health Organization [16], International Code of Marketing of Breast-Milk Substitutes [17], the vigorous promotion the benefits of breastfeeding and the encouragement of maternal breastfeeding behavior. However, the global breastfeeding situation of preterm infants is not optimistic. The average breastfeeding rate of preterm infants during hospitalization in developed countries is 13–49% [18, 19]. However, the breastfeeding rate of premature infants in China is far lower than that of in developed countries. Although China has implemented related measures to promote the breastfeeding rate of preterm infants [20], the breastfeeding rate of preterm infants in the NICU is still only 15% [21].

The breastfeeding outcome of premature infants separated from their mothers has not been investigated in detail in Shanghai in recent years. Premature infants are more likely to face lactation-related problems. This is the first cohort study to investigate factors of exclusive breastfeeding rate and duration of preterm infants separated from their mothers after discharge in Shanghai. The aim of this paper was to investigate the exclusive breastfeeding rate at different stages specifically, at 1 month, 3 months and 6 months after discharge and analyze its determinants of exclusive breastfeeding rate at 6 month and exclusive breastfeeding duration and to improve breastfeeding outcomes after discharge of preterm infants.

## Methods

### Recruitment and sampling

This longitudinal cohort study included 500 premature infants admitted to the NICU and their mothers in a local maternity and special infant hospital in Shanghai. The inclusion criteria for the sample were as follows: women’s delivery gestational age less than 37 weeks; capable of reading and communicating, as well as informed and willing engagement in the research; the premature infants living in the NICU, The exclusion criteria were primiparas who took oral sleeping pills, had a history of mental illness, or those refusing to breastfeed.

The sample size was obtained by cohort formula as follows [22]:

$$\mathrm{n}={\left[{z}_{\alpha}\sqrt{2\overline{p}\overline{\mathrm{q}}}+{z}_{\beta}\sqrt{p_0{q}_0+{p}_1{q}_1}\right]}^2p/{\left({p}_1-{p}_0\right)}^2$$

In the formula, p and q represent the expected incidence rates of the exposed and control groups respectively, pq represents the mean of the incidence rates of the two groups, q = 1-p, α = 0.1, β = 0. 1 [10] and Z is obtained by checking the table Zα = 2.576, Zβ = 1.282 [14]. It is estimated that the breastfeeding rate of preterm infants in the NICU is 15% [21]. Considering the longitudinal study and loss to follow up, we expanded the sample size by 20%. The final sample size was 500.

### Study design and the survey

#### Research instrument

From September 2018 to September 2019, a convenience sample method was utilized to choose primiparous mothers who met the inclusion exclusion criteria in a local maternal and infant-specialized hospital in Shanghai.

On the day of discharge, the socio-demographic characteristics, breastfeeding knowledge questionnaire [23], breastfeeding self-efficacy scale [24]. Edinburgh postpartum depression scale [25] and family support questionnaire [26] were examined and anonymously returned. The findings were kept private and only the research team knew about them. These details were gathered during a face-to-face interview in the hospital shortly after the baby was born. Because the sociodemographic characteristics only marginally evolved over the course 6 months, these data were collected only once.

#### The socio-demographic characteristics

At discharge, the researchers gathered the following parental data: age, education level, occupation, residency,family income, mode of delivery, pregnancy complications, payment type of expenses,whether to live with husbands during perinatal period, planned pregnancy, husband’s attitude towards breastfeeding, prenatal working status, length of stay, plan to work after maternity leave, estimate breastfeeding duration, whether to visit NICU babies,whether to deliver milk to the infants in NICU, maternal lactation experiences(number of removing the breast milk by hand per day, time of each removing the breast milk by hand, methods of removing breast milk during the period of maternal and infant separation) and infant characteristics were collected from the medical records: gender, birth weight, APGAR score, type of feeding in NICU(including exclusive breastfeeding, partial breastfeeding and formula).

The definitions of variables

**Table:** 

variables	definitions
planned pregnancy	defined as the level of readiness of planning for parenthood, divided into prepared, unprepared and fully prepared
pregnancy complications	defined as pregnancy combined with surgical disease
maternal lactation experiences	Maternal lactation behavior during hospitalization, including number of removing the breast milk by hand per day, time of each removing the breast milk by hand, methods of removing breast milk during the period of maternal and infant separation
exclusive breastfeeding	the infant receives only breast milk. no other liquids or solids are given – not even water – with the exception of oral rehydration solution, or drops/syrups of vitamins, minerals or medicines.
partial breastfeeding	you breastfeed for some feedings and supplement with formula for others
formula	liquid food mixture that is fed to babies instead of mother ‘s milk.
breastfeeding duration	breastfeeding duration was calculated using information provided by mothers. Women who were identified as having ever breastfed, not presently breastfeeding, never breastfed, inconsistent, or didn’t know were removed from the variable.

### Breastfeeding knowledge questionnaire [23]

The questionnaire was designed by the researcher after referring to domestic and international literature to measure the knowledge of breastfeeding among primiparous mothers. The questionnaire consists of 17 items, covering both the benefits of breastfeeding and breastfeeding skills. Each item was scored 1 point for a correct answer and the total score was 0–17, with higher scores indicating more maternal breastfeeding knowledge. The content validity index (CVI) of the questionnaire is 0.9 l.

### Breastfeeding self-efficacy scale short form [24]

The Hong Kong Chinese Version of the breastfeeding self-efficacy scale short form is a basic tool for assessing maternal breastfeeding self-efficacy in China. The Breastfeeding Self-Efficacy Brief Scale, which consists of 14 independent items to measure mothers’ confidence in breastfeeding, was translated into a Hong Kong Chinese version by Wan-Yin. All of the items are favorable and they are graded on a 5-point Likert scale. The version of the Breastfeeding Self-Efficacy Scale had a Cronbach’s alpha of 0.941.

### Edinburgh postpartum depression scale [25]

The Edinburgh Postnatal Depression Scale developed by Lee et al. was used in this study to investigate postnatal depression in mothers separated from their infants. The Edinburgh Postnatal Depression Scale was Chineseized and measured by Lee in 1998. The scale consists of 10 domains, covering mood, pleasure, self-blame, anxiety, fear, insomnia, coping skills, sadness, crying and self-harm. The description of each item was divided into 4 levels and scores of 0 (never), 1 (occasionally), 2 (often) and 3 (always) were given according to the severity of the symptoms. The total scores of the ten items were 0–9 (no postpartum depression), 10–13 (mild postpartum depression) and ≥ 14 designated as severe postpartum depression and the scale had an internal consistency reliability of 0.76 and content validity of 0.93.

### Family support questionnaire [26]

The Breastfeeding Family Support Questionnaire developed by Zhu Xiu in 2013 was used to measure family support for breastfeeding. The structure of the questionnaire included two factors: psychological support and behavioral support. Items 1–7 belonged to psychological support and items 8–9 belonged to behavioral support. All items are self-report test questions and multiple-choice items are scored on a 4-point scale (1 to 4). items 2 to 5 are counter scores. The family support score is the average score of the 9 items. A score of 1 indicates a low level of family support, a score of 2 ~ 3 indicates an intermediate level of family support and a score of 4 indicates a high level of family support. The questionnaire has good reliability with a Cronbach’s alpha reliability coefficient of 0.886.

### Data collection

#### Questionnaire survey

With the consent and support of relevant departments of the hospital, surveys were conducted in a local maternity and infant hospital in Shanghai from September 2018 to September 2019. During hospitalization, the primiparas and infants who met the inclusion and exclusion criteria were recruited. On the day of discharge, the researchers explained the purpose and process of the study to the participants and collected the characteristics of mothers and their families and administered the breastfeeding knowledge questionnaire, breastfeeding self-efficacy scale, Edinburgh postpartum depression scale and family support scale. The returned proportion of questionnaires was 100%. If any missing or wrong option was found, relevant content was required to be filled in later.

#### The follow-up process

The follow-up event was the breastfeeding outcomes of premature infants separated from their mothers; the discharge time of premature infants was recorded in their medical history and the follow-up survey was conducted with the day of discharge as the starting point. The results of questionnaires were received by telephone or WeChat at 1 month, 3 months and 6 months after premature infants were discharged from the hospital. The endpoint of observation; breastfeeding outcomes after 1 month, 3 months and 6 months as well as exclusive breastfeeding duration were taken as variables. Finally, incomplete data related to weaning and discontinuation of breastfeeding as well as those who were lost during the follow-up were excluded.

### Statistical analysis

SPSS statistics 24 (IBM, Armonk, NY, USA) was used for data analysis. For regularly distributed variables, a Student’s t-test with mean and standard deviation was utilized. The Chi-square test was used to demonstrate differences in proportions of dichotomous variables and one-way repeated measures ANOVAs were performed for maternal breastfeeding knowledge, self-efficacy, postpartum depression and family support at different stages to explore trends at different stages with a spherical test *p*-value > 0.05. We utilized adjusted logistic regression analysis to look at the influence of the separation on exclusive breastfeeding at 6 months postpartum. Exclusive breastfeeding was compared to partial/no breastfeeding in the logistic regression analysis, which meant that partial and no breastfeeding were pooled into one category. Estimates were obtained using the Kaplan-Meier method The time variable was exclusive breastfeeding duration and participants ceasing breastfeeding were considered an event. The socio-demographic characteristics,breastfeeding knowledge, self-efficacy, postpartum depression and family support scores were grouped and assigned and all were included in the Kaplan-Meier method for univariate analysis; censored data included loss to follow up on the day of survey termination, still breastfeeding, or interrupted breastfeeding for some reason. The researchers utilized cox proportional hazard regression to see if the preterm infants were at risk of breastfeeding failure during the first 6 months following delivery. HR stands for the likelihood of breastfeeding in Cox regression analysis.

## Results

**Fig. 1:**
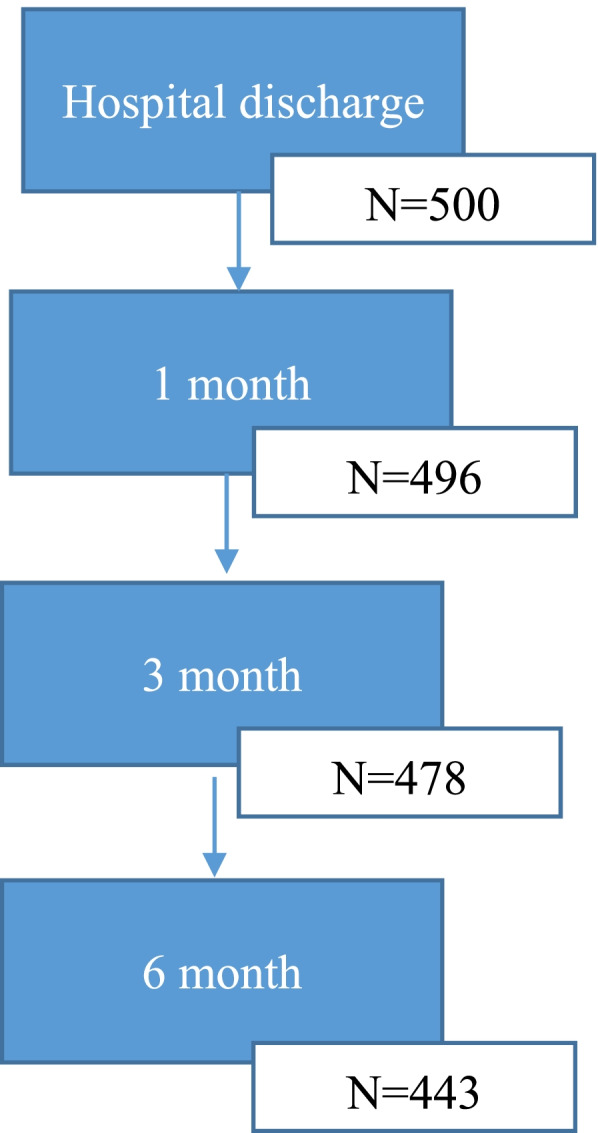
Flowchart

**Fig. 2:**
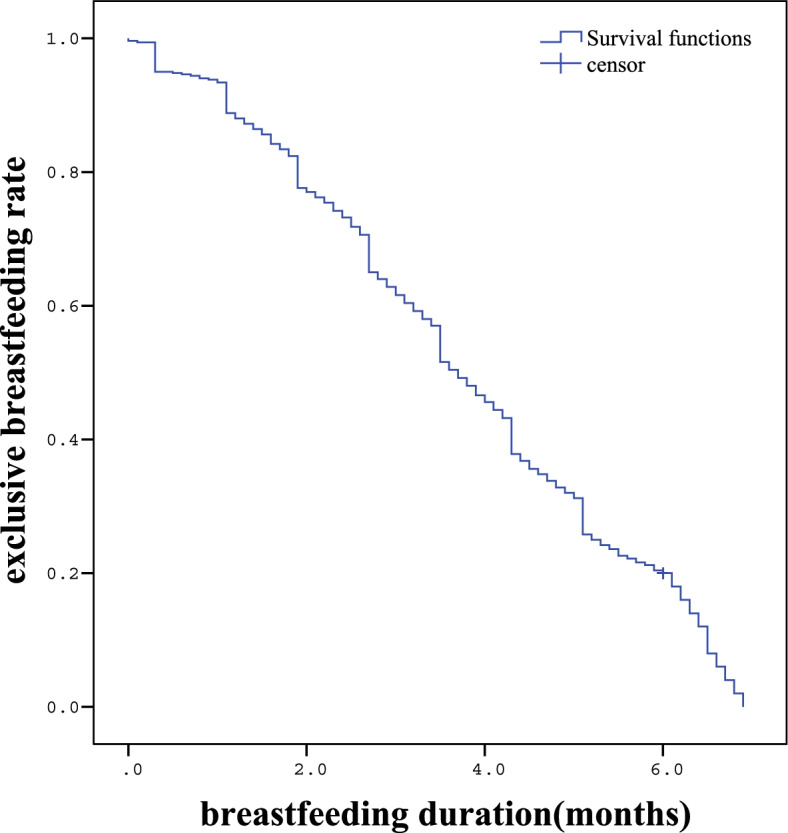
Breastfeeding duration in the whole cohort study. Kaplan-Meier estimates with 95% confidence bands

The initial sample consisted of 500 infants included in the study at birth. Among all preterm infants, 496 infants (99.2%) were followed-up with after 1 month and 478 infants (95.6%) were followed-up with after 3 months. Finally, 443 infants (88.6%) were followed-up with until 6 months later. The average exclusive breastfeeding duration was (3.69 ± 1.80) months and the longest one was 6.90 months. A flowchart with the recruitment and follow-up data appears in Figs. 1 and 2.

### The socio-demographic characteristics

In this study, 500 cases of puerperal and preterm infants were collected. The mean age of mothers was 30.41 years (standard deviation (SD) 4.64 years). In total, 338 mothers had attended an undergraduate program. More than 90% of women were employed during the pregnancy period and 433 mothers lived in the city. More than 40% of families had an income of more than 20,000 RMB monthly,274 patients stayed in the hospital for more than 96 h and 393 puerperal had medical insurance; Additionally, more than 90% of women lived with their husbands during the perinatal period Among the mothers, 412 worked before delivery,411 women decided to continue working after the maternity leave, 85.6% women planned to conceive, 349 women expected to breastfeed for at least 6 to 12 months and 300 women removed the breast milk by hands or pumped the breast, The results showed that 69.6% of women pumped the breast less than eight times per day, 60% women pumped the breast for 30 to 60 min every time;273 mothers or husbands visited the preterm infants during hospitalization and 47.8% women delivered the breast milk to their newborns in the NICU.

The mean age of husbands was 32.41 years (standard deviation (SD) 1.36 years). Two hundred seventy-nine husbands had a bachelor’s degree; and 85.6% of husbands planned to have a baby; they hoped that the exclusive breastfeeding duration would be 6 to 12 months; and 427 husbands’ attitudes towards breastfeeding reached 9–10 points.

Of the sample, 256 infants were male and 36.8% weighed between 2000 and 2500 g at birth and 308 preterm infants had a APGAR score of 9–10. More than 50% of preterm infants were fed formula in the NICU (Table 1). Due to the NICU environment and its surrounding medical devices, mothers were not permitted to breastfeed their babies directly in the NICU and breast milk was usually expressed by the mother and sent to the NICU. The nurses provided breastfeeding evaluation and guidance based on the lactation diary during breast milk collection and encouraged mothers to keep a breastfeeding diary when they were separated from their babies.

**Table 1:** Parental and preterm infants characteristics (*n* = 500)

Variable	Category	N	%
Maternal Age	18 ~ 25	35	7.0
(mean = 30.41)	26 ~ 30	172	34.4
	31 ~ 34	243	48.6
	≥35	50	10.0
Maternal educational level	Primary studies	15	3.0
	Secondary studies	49	9.8
	Undergraduate studies	338	67.6
	Postgraduate studies	98	19.6
Maternal occupation	Working	494	98.8
	Unemployed	6	1.2
Residency	City	433	86.6
	Countryside	67	13.4
Family income (RMB)	≤5000	39	7.8
	5001 ~ 10,000	79	15.8
	10,001 ~ 15,000	91	18.2
	15,001 ~ 20,000	89	17.8
	20,001 ~ 25,000	126	25.2
	25,001 ~ 30,000	36	7.2
	30,001 ~ 35,000	28	5.6
	≥35,001	12	2.4
Mode of delivery	Vaginal	221	44.2
	Cesarean	279	55.8
Gestational week	28 ~ 32^+ 6^	43	8.6
	33 ~ 34^+ 6^	49	9.8
	35 ~ 36^+ 6^	408	81.6
Labour Complication	Yes	252	50.4
	No	248	49.6
Payment type of expenses	Medical insurance	393	78.6
	Self-paid	92	18.4
	Other	9	1.8
	Unclear	6	1.2
Live with husbands during Perinatal period	Yes	487	97.4
	No	13	2.6
Prenatal work	Yes	412	82.4
	No	88	17.6
Length of hospital stay (hour)	36 ~ 48	26	5.2
	49 ~ 72	102	20.4
	73 ~ 96	98	19.6
	≥97	274	54.8
Plan to work after maternity leave	Yes	411	82.2
	No	89	17.8
Planned pregnancy	Prepared	328	65.6
	unprepared	72	14.4
	fully prepared	100	20.0
Estimated breastfeeding time (month)	<6	100	20.0
	6 ~ 12	349	69.8
	12 ~ 24	49	9.8
	>24	2	0.4
Methods of removing breast milk during the period of mother and infant seperation	By hand	63	12.6
	Breast pump	137	27.4
	By hand+ Breast pump	300	60.0
Number of removing the milk by hand per day	<8	348	69.6
	8 ~ 12	149	29.8
	>12	3	0.6
Time of each removing the milk by hand	<30	158	31.6
	30 ~ 60	300	60.0
	>60	42	8.4
Visit NICU newborns	Yes	273	54.6
	No	227	45.4
Deliver milk to the infants in NICU	Yes	239	47.8
	No	261	52.2
Husband Age (mean = 32.95)	18 ~ 25	15	3.0
	26 ~ 30	150	30.0
	31 ~ 34	195	39.0
	≥35	140	28.0
Husband’s education. Level	Primary studies	13	2.6
	Secondary studies	67	13.4
	Undergraduate degree	279	55.8
	Postgraduate studies	141	28.2
Planned pregnancy	Yes.	428	85.6
	No.	72	14.4
Husband’s attitude towards breastfeeding.	5	7	1.4
	6	11	2.2
	7	1	0.2
	8	54	10.8
	9	31	6.2
	10	396	79.2
Infant gender	Male	256	51.2
	Female	244	48.8
Weight at birth(g)	<1500	28	5.6
	1500 ~ 2000	42	8.4
	2001 ~ 2500	128	25.6
	2501 ~ 3000	184	36.8
	≥3001	118	23.6
Apgar score	≤6	13	2.6
	7 ~ 8	179	35.8
	9 ~ 10	308	61.6
Type of feeding in NICU	Exclusive breastfeeding	38	7.6
	Partial breastfeeding	195	39.0
	Formula	267	53.4

About 19% of preterm infants were breastfed after one-month after discharge from the hospital and the percentage dropped to 17 and 10% at 3 and 6 months after discharge, respectively (Fig. 2).

### Questionnaires survey

Table 2 shows that maternal breastfeeding knowledge was (13.63 ± 2.93) at the hospital and decreased to (12.67 ± 3.72) after 6 months; maternal breastfeeding self-efficacy was (3.25 ± 0.77) at the hospital and increased to (3.34 ± 1.04) in 6 months; maternal postpartum depression was (16.63 ± 2.83) and decreased to (13.06 ± 1.86) after 6 months; maternal family support was (2.94 ± 0.40) and increased to (3.06 ± 1.11) after 6 months. Through the ANOVA, the following results were obtained over time, there was a significant difference between maternal breastfeeding self-efficacy and postpartum depression (*P* < 0.05); the longer the time period was, the more did breastfeeding self-efficacy show an upward trend and the more did the degree of postpartum depression decrease. Having a newborn in the NICU was linked to severe anxiety, despair and exhaustion in parents. This was also described as distressing and parents reported feeling overburdened and disoriented in the interim between being and not being parents.

**Table 2:** Maternal Breastfeeding knowledge, Breastfeeding self-efficacy, Postpartum depression and Family support at birth,1,3,6 months

Factor	At hospital ($\overline{x}$ *± s*)	1 month ($\overline{x}$ *± s*)	3 months ($\overline{x}$ *± s*)	6 months ($\overline{x}$ *± s*)	Within-group factors	
					*F*	*P*
Breastfeeding knowledge	13.63 ± 2.93	13.05 ± 3.68	12.37 ± 4.37	12.67 ± 3.72	2.403	0.108
Breastfeeding self-efficacy	3.25 ± 0.77	3.29 ± 1.04	3.34 ± 1.05	3.34 ± 1.04	55.228	<0.001
Postpartum depression	16.63 ± 2.83	13.28 ± 2.14	13.15 ± 2.01	13.06 ± 1.86	19.930	<0.001
Family support	2.94 ± 0.40	3.03 ± 1.15	3.01 ± 1.15	3.06 ± 1.11	1.624	0.226

*F* Fish statistic, *P P* value

### Factors associated with exclusive breastfeeding rate at 6 month

Type of delivery, gestational age, deliver milk to the infants in NICU, APGAR score, type of feeding in NICU, breastfeeding knowledge, breastfeeding self-efficacy, postpartum depression and maternal family support were associated with breastfeeding outcome 6 months after discharge of preterm infants (*P* values < 0.05) (Table 3).

**Table 3:** Univariate analysis of exclusive breastfeeding rate at 6 months (*N* = 443)

Variable	6 follow-up Numbers(Percentages%)	exclusive breastfeeding numbers(Percentages%)	*H/χ*^*2*^	*P*
Maternal Age			3.247^a^	0.918
18 ~ 25	31(7.0%)	5(16.1%)		
26 ~ 30	151(34.1%)	19(12.6%)		
31 ~ 34	217(49.0%)	23(10.6%)		
≥ 35	44(9.9%)	5(11.4%)		
Maternal educational level			1.218 ^a^	0.976
Primary studies	12(2.7%)	1(8.3%)		
Secondary studies	45(10.2%)	4(8.9%)		
Undergraduate studies	300(67.7%)	36(12.0%)		
Postgraduate studies	86(19.4%)	11(12.8%)		
Maternal occupation			4.598^a^	0.970
Employment	437(98.6%)	51(11.7%)		
Unemployed	6(1.4%)	1(16.7%)		
Residency			2.041^a^	0.728
Urban	382(86.2%)	43(11.3%)		
Rural	61(13.8%)	9(9.8%)		
Family income(RMB)			3.555^a^	0.895
<5000	33(7.4%)	3(9.1%)		
5000 ~ 10,000	68(15.3%)	9(13.2%)		
10,000 ~ 15,000	75(16.9%)	7(9.3%)		
15,000 ~ 20,000	77(17.4%)	10(13.0%)		
20,001 ~ 25,000	124(28.0%)	7(5.6%)		
25,001 ~ 30,000	32(7.2%)	5(15.6%)		
30,001 ~ 35,000	25(5.6%)	3(12.0%)		
≥ 35,001	9(2.0%)	8(88.9%)		
Type of delivery			6.166^a^	0.046*
Vaginal	196(44.2%)	31(15.8%)		
Cesarean	247(55.8%)	21(8.5%)		
Gestational age (week)			15.401^a^	0.004*
28 ~ 32^+ 6^	40(9.0%)	3(7.5%)		
33 ~ 34^+ 6^	44(9.9%)	4(9.1%)		
35 ~ 36^+ 6^	359(81.0%)	45(12.5%)		
Labor Complication			4.085^a^	0.982
No	215(48.5%)	26(12.1%)		
Yes	228(51.5%)	26(11.4%)		
Payment type of expenses			4.618^a^	0.594
Medical insurance	349(78.8%)	41(11.7%)		
Self-paid	79(17.8%)	8(10.1%)		
Other	6(1.4%)	1(16.7%)		
Unclear	9(2.0%)	2(22.2%)		
Live with husbands during Perinatal period			2.180^a^	0.336
Yes	431(97.3%)	49(11.4%)		
No	12(2.7%)	3(25.0%)		
Prenatal work			0.069^a^	0.966
Yes	371(83.7%)	44(11.9%)		
No	72(16.3%)	8(11.1%)		
Length of hospitalization(h)			10.795^a^	0.095
36 ~ 48	18(4.1%)	2(11.1%)		
49 ~ 72	96(21.7%)	15(15.8%)		
73 ~ 96	81(18.3%)	8(9.9%)		
>96	248(56.0%)	27(10.9%)		
Plan to work after maternity leave			0.188 ^a^	0.943
Yes	366(82.6%)	43(11.7%)		
No	77(17.4%)	9(11.7%)		
Planned pregnancy			3.181^a^	0.528
Yes	67(15.1%)	19(28.4%)		
No	376(84.9%)	67(17.8%)		
Estimated breastfeeding time(month)			1.286^a^	0.972
≤ 6	77(17.4%)	7(9.1%)		
6 ~ 12	322(72.7%)	41(12.7%)		
12 ~ 24	43(9.7%)	4(9.3%)		
>24	1(0.2%)	0(0.0%)		
Methods of removing breast milk during the period of mother and infant seperation			2.500^a^	0.645
By hand	54(12.2%)	7(13.0%)		
Breast pump	129(29.1%)	16(12.4%)		
By hand+ Breast pump	260(58.7%)	29(11.2%)		
Number of removing the milk by hand per day			2.880^a^	0.578
<8	309(69.8%)	32(10.4%)		
8 ~ 12	132(29.8%)	20(15.2%)		
>12	2(0.5%)	0(0.0%)		
Time of each removing the milk by hand			0.163^a^	0.997
<10	135(30.5%)	16(11.9%)		
10 ~ 30	268(60.5%)	32(11.9%)		
>30	40(9.0%)	4(10.0%)		
Visit NICU newborns			0.317^a^	0.853
Yes	243(54.9%)	28(11.5%)		
No	200(45.1%)	24(12.0%)		
Deliver milk to the infants in NICU			11.429^a^	0.003*
Yes	236(56.6%)	39(16.5%)		
No	207(46.7%)	13(6..3%)		
Husband Age(years)			2.527^a^	0.960
18 ~ 25	15(3.4%)	2(13.3%)		
26 ~ 30	131(29.6%)	17(13.0%)		
31 ~ 34	169(38.1%)	17(10.1%)		
≥ 35	128(28.9%)	16(12.5%)		
Husband’s education. Level			4.120^a^	0.660
Primary studies	10(2.3%)	1(10.0%)		
Secondary studies	65(14.7%)	8(12.3%)		
Undergraduate studies	247(55.8%)	28(11.3%)		
Postgraduate studies	127(28.7%)	15(11.8%)		
Husband’s Planned pregnancy			5.102^a^	0.531
Yes	66(14.9%)	10(15.2%)		
No	377(85.1%)	42(11.1%)		
Husband’s attitude towards breastfeeding.			5.654^a^	0.686
5	6(1.4%)	1(16.7%)		
6	11(2.5%)	1(9.1%)		
7	0(0.0%)	0(0.0%)		
8	46(10.4%)	6(13.0%)		
9	25(5.6%)	2(8.0%)		
10	355(80.1%)	42(11.8%)		
Infant gender			0.219^a^	0.896
Male	221(49.9%)	27(12.2%)		
Female	222(50.1%)	25(11.3%)		
Weight at birth(g)			4.879^a^	0.770
<1500	22(5.0%)	3(5.8%)		
1500 ~ 2000	36(8.1%)	4(11.1%)		
2000 ~ 2500	118(26.6%)	9(7.6%)		
2500 ~ 3000	50(11.3%)	6(12.0%)		
>3000	217(49.0%)	30(13.8%)		
Apgar score			17.940^a^	0.006*
≤ 6	12(2.7%)	1(8.3%)		
7	38(8.6%)	2(5.3%)		
8	107(24.2%)	5(4.7%)		
9 ~ 10	286(64.6%)	44(15.4%)		
Type of feeding in NICU			13.346^a^	0.010*
Exclusive breastfeeding	41(9.3%)	11(26.8%)		
Partial breastfeeding	185(41.8%)	21(11.4%)		
Formula	217(49.0%)	20(9.2%)		
Breastfeeding knowledge level			58.991^a^	0.000*
Higher score(>14)	232(52.4%)	42(18.1%)		
lower score(≤14)	211(47.6%)	10(4.7%)		
Breastfeeding self-efficacy levels			58.050^a^	0.000*
Higher score(≤3)	249(56.2%)	10(4.0%)		
lower score(>3)	194(43.8%)	42(80.8%)		
Postnatal depression			87.614a	0.000*
No depression(≤9)	0(0.0%)	0(0.0%)		
Mild depression(10 ~ 13)	222(50.5%)	38(17.1%)		
Major depression(≥14)	221(49.9%)	14(6.3%)		
Maternal family support			71.828^a^	0.000*
lower score(≤3)	220(49.7%)	10(4.5%)		
Higher score(>3)	223(50.3%)	42(18.8%)		

Note: a is the H value; * is the *P* value < 0.05

In the multivariate logistic regression model established with exclusive breastfeeding at 6 months post-discharge for preterm infants separated from mothers as the dependent variable, the factors that entered the equation were breastfeeding delivery or not during hospitalization, infant APGAR score, feeding style of preterm infants in NICU, breastfeeding knowledge, breastfeeding self-efficacy, postpartum depression level and maternal family support status. The final multifactorial outcome analysis that entered the equation showed that type of delivery, gestational age, maternal family support were significant factors influencing the exclusive breastfeeding rate at 6 months after discharge for preterm infants, as detailed in Table 4.

**Table 4:** Results of multiple logistic regression analysis of exclusive breastfeeding rate at 6 months

Variable	Unadjusted OR (95%CI)	p	Adjusted OR (95%CI)	p
Type of delivery	1.727(0.697, 4.280)	0.023	1.564(0.513, 3.116)	0.015
Gestational age	0.519(0.112, 2.407)	0.040	0.612(0.236, 3.418)	< 0.001
Maternal family support	76.923(7.752, 100.781)	0.000	68.125(6.359, 98.452)	< 0.001

*OR* Odds ratio, *CI* Confidence Interval

### Factors associated with exclusive breastfeeding duration

**Fig. 3:**
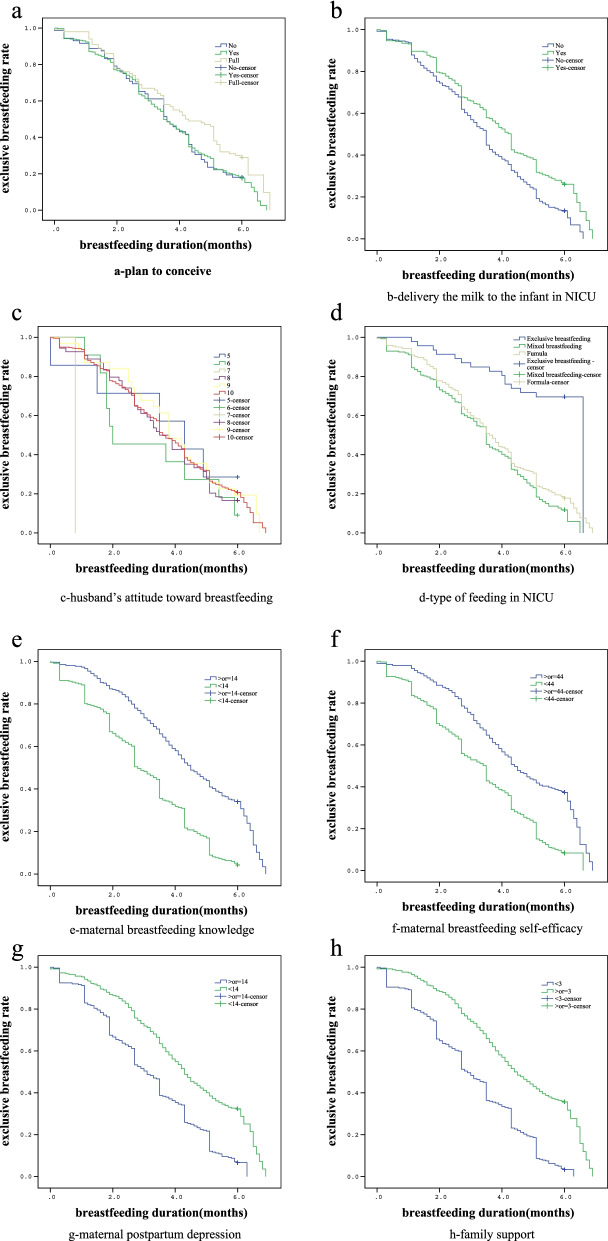
Duration of breastfeeding according to (**a**) plan to conceive, (**b**) Deliver milk to the infants in NICU, (**c**) Husband’s attitude towards breastfeeding, (**d**) Type of feeding in NICU, (**e**) Breastfeeding knowledge level, (**f**) Breastfeeding self-efficacy, (**g**) Postpartum depression, (**h**) Family support

Table 5 shows the results from the Kaplan-Meier curve. The maternal and family characteristics, breastfeeding knowledge, breastfeeding self-efficacy, postpartum depression and family support were included in the Kaplan-Meier method for univariate analysis. There were significant differences in the exclusive breastfeeding duration among the levels of psychological preparation for pregnancy, delivering milk to the infants in the NICU, the attitude of spouses towards breastfeeding, type of feeding in the NICU, maternal breastfeeding knowledge, breastfeeding self-efficacy, maternal postpartum depression and family support (P<0.05). The data are shown in Table 5 and Fig. 3.

**Table 5:** Factors associated with exclusive breastfeeding duration: results obtained by Kaplan Meier curve

Variable	The type of breastfeeding outcome			Median.	*95%CI*	Kaplan-Meier	
	censoring	Breastfeeding(N)	N%.	(Month)		*χ2*	*P*
Planned pregnancy						7.264	0.026
Unprepared	13	59	81.944	3.6	2.98,4.22		
Prepared	51	277	84.451	3.5	3.20,3.80		
Fully prepared	26	74	74	4.2	3.45,4.95		
Deliver milk to the infants in NICU						15.346	<0.001
Yes	62	199	76.245	4.3	4.06,4.54		
No	28	211	88.285	3.5	3.27,3.73		
Husband’s attitude towards breastfeeding.						16.649	0.005
5	2	5	71.429	4.3	2.25,6.35		
6	1	10	90.909	2.0	0.00,4.05		
7	0	1	100.000	0.8	–		
8	9	45	83.333	3.5	2.87,4.13		
9	4	27	87.097	3.8	3.18,4.42		
10	74	322	81.313	3.6	3.33,3.87		
Type of feeding in NICU						45.371	<0.001
Exclusive breastfeeding.	31	15	39.474	6	–		
Mixed feeding.	23	189	89.231	3.5	3.25,3.75		
Formula	36	206	82.772	3.7	3.29,3.92		
Breastfeeding knowledge level (points)						82.015	<0.001
≥ 14	80	184	69.697	4.5	4.08,4.93		
<14	10	226	95.763	2.8	2.47,3.13		
Breastfeeding self-efficacy (points)						53.184	<0.001
≥ 44	66	135	67.164	4.4	3.94,4.86		
<44	24	275	91.973	3.5	3.16,3.84		
Postpartum depression (points)						53.221	<0.001
≥ 14	75	185	92.5	4.3	3.95,4.65		
<14.	15	225	75	3.1	2.85,3.35		
Family support (points)						86.196	<0.001
≥ 3.	83	175	67.829	4.4	3.954.85		
<3 .	7	235	97.107	3.9	2.66,3.14		

*χ2:*Chi-square test; *P p*-Value, *CI* Confidence Interval

A significant single factor was included in the cox regression and the results showed that the more sufficient the degree of psychological preparation for planned pregnancy, the lower the risk of weaning and the longer the exclusive breastfeeding duration. in the multivariate analysis (HR:0.681;95%CI:0.531,0.873).Women who delivered milk to the NICU during hospitalization had a lower risk of weaning than those who did not and also breastfed the newborns longer(HR:0.681;95% CI:0.412, 2.288). Premature infants who were fed formula in the NICU had a higher risk of weaning after discharge and were more prone to stop breastfeeding (HR:1.221; 95% CI 0.128,1.381). Women with higher levels of family support had a lower risk of weaning their preterm infants after discharge from the hospital and were more likely to keep breastfeeding (HR: 0.561; 95% CI 0.004,2.428). The data are shown in Table 6.

**Table 6:** factors associated with the risk of discontinuing exclusive breastfeeding which is the outcome event. Risks for ceasing breastfeeding in final model using Cox regression analyses with hazard ratios (HR) and 95%confidence intervals (95%CI)

Variable	*Β*	*HR*	*95%CI*	*Se*	*z*	*P*
Planned pregnancy	−0.385	0.681	0.531,0.873	0.127	9.166	0.002
Milk delivery during hospitalization	−0.586	0.797	0.412,2.288	0.123	22.706	<0.001
Artificial feeding of premature infants in NICU	1.509	1.221	0.128,1.381	0.278	29.512	<0.001
High Level of family support	−0.445	0.561	0.004,2.428	0.225	3.904	0.048

*Β* Regression Coefficient, *HR* risk ratio; *CI* Confidence Interval; *Se* standard error; *z* Wald Statistics

*P p*-Value

## Discussion

### Low exclusive breastfeeding rate in 6 months

According to the China Development Outcome of Child (2011–2020), the percentage of babies aged 0 to 6 months who are exclusively breastfed would reach 50% or higher [27]. In this study, the exclusive breastfeeding rate of preterm infants was 19.0% in the first month, 17.2% in the third month and only 10.4% in the sixth month after discharge from the hospital. The exclusive breastfeeding rate for preterm children rapidly fell after discharge and was significantly lower than the 36.18% exclusive breastfeeding rate for full-term infants in the first 6 months of life [28]. Preterm children may be born with severe respiratory distress, hypoglycemia and hypothermia as a result of immature physiological development, leading in maternal-infant separation and delayed breastfeeding initiation [2].Second, sucking-swallowing-respiratory function does not become gradually coordinated until around 37 weeks [3]. In addition, preterm newborns may be unable to create appropriate negative pressure while sucking due to underdevelopment of the cheek fat bed [29]. During maternal-infant separation, maternal frequent hand pumping is required to ensure that the ducts are unblocked and a lack of adequate breastfeeding knowledge and skilled lactation can result in breast swelling and acute mastitis, the mothers have to stop breastfeeding after medication, directly leading to breastfeeding failure after the preterm infant is discharged. Breastfeeding success after preterm infant discharge is inextricably linked to frequent sucking, skin-to-skin contact, early perception of the infant’s physiological state and effective response adjustment. Exclusive breastfeeding following preterm infant discharge is currently unfavorable and healthcare providers should keep this in mind when investigating the causes of exclusive breastfeeding failure.

### Analysis of the time-varying covariates of maternal breastfeeding knowledge, self-efficacy, postpartum depression and family support

Within 6 months following delivery, the mean scores for maternal breastfeeding knowledge varied from (12.37 ± 4.37) to (13.63 ± 2.93), which was lower than the survey by Layal Hamze et al. [23]. Inadequate breastfeeding education and support after discharge may be linked to low maternal breastfeeding knowledge.

After discharge, the mother’s breastfeeding self-efficacy improved steadily, the baby’s continual interaction with her increased the mother’s breastfeeding self-efficacy and the mother’s increasing willingness to breastfeed made it simpler for her to keep to breastfeeding. Breastfeeding self-efficacy is regarded to be a factor of breastfeeding duration and is changing [30]. Creating strategic plans and setting targets to assist women who are separated from their infants in enhancing their nursing self-efficacy is crucial.

Postpartum depression (PPD) is a perinatal form of major depressive disorder (MDD) and affects approximately 500,000 women annually in the US (prevalence 10–15%) [31], while in China, the prevalence were about 23.5% [32]. In the research, maternal PPD levels increased significantly during mother-infant separation, but steadily decreased when the preterm infant was discharged from the NICU. Our findings were much higher than the prevalence reported in the literature, maybe due to premature births and maternal and infant separation. The physical health of a premature infant will have a substantial impact on the mother’s emotional health.

The level of maternal family support was modest in the study. Having maternal support from family, friends and health care providers can help you establish successful breastfeeding [33]. A mother’s decision to breastfeed her infant is influenced by her family [34]. Family support for breastfeeding should be strengthened in women who have separated their infants from their mothers and in preterm neonates. Because most Chinese families only have one child due to the country’s family planning policy, the child naturally becomes the family’s focal point. The majority of family members’ preparation for a baby’s birth is material and lacks information. After the premature baby is discharged from the hospital, family members’ lack of knowledge about breastfeeding will impede mother breastfeeding.

### Factors associated with exclusive breastfeeding rate at 6 month

Smaller gestational age was found to be an unfavorable factor in the exclusive breastfeeding rate of preterm infants 6 months after discharge from the hospital, which was consistent with Perrella’s research [35]. Because most preterm infants need to be transported to the NICU right after birth and are separated from their mothers, delayed breastfeeding initiation occurs [23] and the younger the gestational age, the longer the hospital stay. In addition, preterm infants less than 34 weeks of gestational age cannot establish effective swallowing and sucking, necessitating nasal feeding. In certain cases, preterm infants are discharged with indwelling nasal feeding tubes, despite their inability to attain complete oral feeding. Mothers must express their breast milk by hand or use a breast pump to maintain lactation during this time and perceived inadequate milk supply (PIMS) is one of the leading causes of breastfeeding discontinuation [36]. In this cohort study, maternal-infant separation was found to be most common in late preterm newborns (LPT)(34^+ 0^–36^+ 6^), who had a low exclusive breastfeeding rate after discharge from the hospital, only 45 of 359 (12.5%) late premature infants were exclusively breastfed 6 months after discharge from the hospital, far below the WHO target of 50% exclusive breastfeeding rate at 6 months [27]. Several studies have revealed lower rates of breastfeeding initiation and shorter breastfeeding duration in LPT newborns when compared to term infants [37, 38], despite the recognized short- and long-term effects of LPT births, as well as the mother and baby health benefits of breastfeeding [37]. These differences in breastfeeding could be due to a variety of factors, including maternal medical difficulties, delayed lactogenesis and infant clinical abnormalities. Better breastfeeding success for LPT infants in the NICU could be explained by more support and systematic breastfeeding education in the NICU [37, 39]. Medical personnel should pay special attention to preterm newborns, teach women to monitor daily lactation, keep lactation diaries and intervene as needed to optimize maternal lactation and promote nursing for infants of lower gestational ages.

In the research, type of delivery also influenced the exclusive breastfeeding rate of preterm infants for 6 months after discharge. Compared to cesarean delivery, preterm infants vaginal delivery had a higher exclusive breastfeeding rate after discharge from the hospital(44.2%vs55.8%).Incision pain, postural limitations and delayed lactation following a cesarean section all make these preterm infants breastfeeding more challenging, resulting in a low breastfeeding success rate among cesarean section moms [36]. In addition, women who had a cesarean section showed a lower readiness to breastfeed and experienced more feeding issues and obstacles than women who had a vaginal delivery [36]. Another factor that may have contributed to the high cesarean section rate was a new birth policy enacted in 2013 to encourage families to have two children, which resulted in an increase in the number of older pregnant women who underwent cesarean surgery for a variety of reasons [11].

### Factors associated with exclusive breastfeeding duration

In the research, that exclusive breastfeeding duration was decided by maternal planned pregnancy, maternal and family action (delivery of the breast milk to the infants separated from their mothers during hospitalization), medical intervention (artificial feeding of premature infants in the NICU) and family support. The psychological status of the mothers before pregnancy also had a great impact on the breastfeeding duration. Therefore, the maternal psychological state of preparation for pregnancy should be paid greater attention to [37]. The more psychological preparation for pregnancy a mother had, the lower the risk of weaning in the future. Meanwhile, they were also more aware of the benefits of breastfeeding and were empowered to decide whether to breast feed or not [37].

The delivery of breast milk by mothers and their families was linked to a higher exclusive breastfeeding rate and longer exclusive breastfeeding duration. According to studies, the lower the premature infant’s birth weight, the longer it takes for them to adjust to direct breastfeeding following discharge. Early breastfeeding termination was usually linked to shorter pregnancy duration and a lower birth weight [40]. The organ system of sucking in preterm newborns became less mature as the gestational week progressed, resulting in poor swallowing and aspiration coordination, necessitating nasal feeding [34]. Premature newborns will be able to start breastfeeding as soon as feasible if breast milk is delivered early to the NICU.

Artificial feeding of premature infants in the NICU has also been established as a factor linked to exclusive breastfeeding duration after discharge, according to our findings. The risk of breastfeeding cessation was raised when neonates were fed formula. Mothers of infants who were breastfed during hospitalization were more likely to continue to direct breastfeed following discharge. In a national study on preterm infant breastfeeding in Denmark, it was discovered that allowing mothers to visit the NICU to care for preterm newborns via cup-feeding or spoon-feeding could lead to premature infants accepting direct nursing sooner after discharge [41]. In China, the traditional NICU was designed as a multi-person room with many warming boxes in a large open space to allow a medical staff to observe multiple infants at the same time and reduce walking distances. Because of the NICU’s closed environment in China, parents are not permitted to visit their children. Premature newborns are typically fed formula, which presents a challenge for mothers who wish to continue breastfeeding their babies after they are discharged from the hospital. Participants reported a sense of intimacy with their preterm infants when feeding, holding and engaging with them. Participants recognized intimate physical contact between the parent and the infant as an important aspect of intimacy in all of these actions. The NICU is more than simply a treatment facility for children; it is also a living environment for them and their families, with a focus on family-centered care. The most recent NICU arrangement in the United States is more equipped to gather the necessities of families in the family room (Single-Family Room, SFR). According to a study [42], SFRs invigorate breastfeeding because of the advancement of breastfeeding training provided to families by clinical professionals, allowing moms to acquire enough expert aid. SFR allowed mothers to be directly identified with truly focusing on their infants. They were also likely to have maternal assurance as a result of it, which aided in the early establishment and maintenance of breastfeeding. Therefore, they were also more likely to gain maternal confidence as a result of it, making it easier to initiate and maintain breastfeeding early on. As a result, it is recommended that the NICU be opened twice or three times a week to increase the contact time between the mother and the preterm infants. In the meantime, mothers should be encouraged to express milk on the cot side, which can relieve the mother’s anxiety and increase the exclusive breastfeeding rate.

### Factors associated with exclusive breastfeeding rate and duration

This study pointed out that maternal family support not only affected the exclusive breastfeeding rate but also the duration. Breastfeeding success is dependent on a supportive family setting. However, the results of this survey revealed that family support for breastfeeding is low, particularly in terms of psychological support. The score of family support during hospitalization was only (2.79 ± 0.37), whereas the score of behavioral support was slightly higher. For example, when asked if “my family believes formula may substitute breast milk,” the overall consensus was that formula could be given instead of breast milk when breast milk was insufficient. It was shown that family members lacked breastfeeding knowledge and they wanted to help mothers in their own way. Therefore, the establishment of a supportive environment was essential to improving breastfeeding outcomes for most families.

The high rate of lost to follow up would probably influence the results. Therefore, every effort was made to reduce the number of lost to follow ups. There were reminders about the follow-up. Defaulted mothers were contacted and given a different date. To overcome the inaccuracy caused by lost to follow ups, we conducted a longitudinal analysis on lost to follow up rates. In the implementation of clinical research, the lost to follow up rate of no more than 20% is generally guaranteed [43]. In this study, which was a longitudinal survey of breastfeeding and maternal related conditions of preterm infants at 1 month, 3 months and 6 months after discharge, the lost to follow up rate was 11.4%, which was within the acceptable range. The socio-demographic features of lost to follow ups were compared to those of preterm infants who completed the follow-up and found no significant differences. In this study we only calculated the minimum sample size based on the cohort study formula, in the future as our sample size increases and our uncertainty decreases, we will have greater precision for breastfeeding follow-up results.

Our study also had some limitations. First, exclusive breastfeeding duration was reported by mothers or their husbands, so there was some room for information bias as some women could have shared information according to social desires more than according to their actual practice. Second, although the sample size was only 500 mother-infant dyads, some categories in the analysis had fewer participants, which made some confidence intervals excessively wide. Finally, due to human and time restrictions, this study only followed women for 6 months after giving birth. In addition, according to the WHO recommendation of exclusive breastfeeding for 6 months after delivery [27]. In future investigations, the duration of longitudinal follow-up could be prolonged. In the Ericson study [9] it was noted that 21% preterm infants (*n* = 49) partially breastfed at 12 months, the overall breastfeeding prevalence would be lower if moms who were not breastfeeding at discharge had been included. This conclusion was consistent with our study. The main strength of our study was that women and preterm infants were prospectively followed homogeneously in a single center committed to breastfeeding practices.

## Conclusions

Using data from our study, we have demonstrated that the exclusive breastfeeding rate was low and breastfeeding duration of premature infants after discharge was short. Therefore, targeted measures should be taken according to the different stages. Specifically, we need to focus on the three system levels of the individual, the family and society, improve maternal breastfeeding knowledge and self-efficacy, pay attention to maternal postpartum emotions, encourage family members to participate in the process of breastfeeding and improve family support as much as possible. Second, to the medical staff, an open neonatal intensive care unit environment should be provided to the parents. Last but not least, on the societal level, breastfeeding support policies should be optimized to improve the breastfeeding rate and prolong the breastfeeding time of premature infants after discharge.

## Data Availability

The questionnaire and datasets used during the current study are available from the corresponding author upon reasonable request.

## Abbreviations

ROP: Retinopathy of prematurity
CI: Confidence interval
NICU: Neonatal intensive care unit
SD: Standard deviation
UOR: Unadjusted odds ratio
